# Lansoprazole promotes cisplatin‐induced acute kidney injury via enhancing tubular necroptosis

**DOI:** 10.1111/jcmm.16302

**Published:** 2021-02-18

**Authors:** Lin Ye, Wanxia Pang, Yanheng Huang, Hongluan Wu, Xiaorong Huang, Jianxing Liu, Shujun Wang, Chen Yang, Qingjun Pan, Huafeng Liu

**Affiliations:** ^1^ Key Laboratory of Prevention and Management of Chronic Kidney Disease of Zhanjiang City Institute of Nephrology Affiliated Hospital of Guangdong Medical University Zhanjiang China; ^2^ Department of Nephrology Maoming People's Hospital Maoming China

**Keywords:** acute kidney injury, cisplatin, inflammation, lansoprazole, necroptosis

## Abstract

Acute kidney injury (AKI) is the main obstacle that limits the use of cisplatin in cancer treatment. Proton pump inhibitors (PPIs), the most commonly used class of medications for gastrointestinal complications in cancer patients, have been reported to cause adverse renal events. However, the effect of PPIs on cisplatin‐induced AKI remains unclear. Herein, the effect and mechanism of lansoprazole (LPZ), one of the most frequently prescribed PPIs, on cisplatin‐induced AKI were investigated in vivo and in vitro. C57BL/6 mice received a single intraperitoneal (i.p.) injection of cisplatin (18 mg/kg) to induce AKI, and LPZ (12.5 or 25 mg/kg) was administered 2 hours prior to cisplatin administration and then once daily for another 2 days via i.p. injection. The results showed that LPZ significantly aggravated the tubular damage and further increased the elevated levels of serum creatinine and blood urea nitrogen induced by cisplatin. However, LPZ did not enhance cisplatin‐induced tubular apoptosis, as evidenced by a lack of significant change in mRNA and protein expression of Bax/Bcl‐2 ratio and TUNEL staining. Notably, LPZ increased the number of necrotic renal tubular cells compared to that by cisplatin treatment alone, which was further confirmed by the elevated necroptosis‐associated protein expression of RIPK1, p‐RIPK3 and p‐MLKL. Furthermore, LPZ deteriorated cisplatin‐induced inflammation, as revealed by the increased mRNA expression of pro‐inflammatory factors including, NLRP3, IL‐1β, TNF‐α and caspase 1, as well as neutrophil infiltration. Consistently, in in vitro study, LPZ increased HK‐2 cell death and enhanced inflammation, compared with cisplatin treatment alone. Collectively, our results demonstrate that LPZ aggravates cisplatin‐induced AKI, and necroptosis may be involved in the exacerbation of kidney damage.

## 1 INTRODUCTION

Cisplatin is a potent first‐line chemotherapy agent for many malignancies, such as lung and ovarian cancers. However, the utility of cisplatin in the clinic is limited mainly because of its nephrotoxicity. It has been reported that 20%‐40% of cancer patients exposed to cisplatin experience acute kidney injury (AKI), which is associated with a significantly increased risk of mortality.^1^ Patients that survive AKI are predisposed to develop chronic kidney disease (CKD).^2^ Since there is a lack of an effective therapeutic approach for cisplatin‐induced AKI prevention and treatment,^3^, ^4^ risk factors that induce or promote AKI should be addressed during cisplatin treatment as carefully as possible.

In clinical practice, cisplatin, a high emetogenic chemotherapy drug, is often used in combination with proton pump inhibitors (PPIs). PPIs are among the most commonly used medications to treat a variety of indications, including gastroesophageal reflux disease and gastroduodenal disease.^5^ Evidence shows that approximately 65% of cancer patients receiving acid‐reducing agents receive PPI treatment for gastroesophageal reflux.^6^ Although PPIs are considered to be highly safe and well‐tolerated, they cause some adverse renal events. Recent large clinical population‐based cohort studies have shown that PPIs are associated with an increased risk of AKI, incident CKD, and progression to end‐stage renal disease in different patient populations compared with that in histamine‐2 (H2) receptor antagonist users or PPI non‐users.^7^, ^8^, ^9^, ^10^ Additionally, PPIs are often overused without reasonable indications or medical prescription, leaving us to question the number of different populations at risk of adverse renal events.^11^ In this regard, we need to evaluate the effects of PPI use under co‐administration with cisplatin.

A retrospective cohort study showed that the combination of PPIs alleviated the nephrotoxicity induced by the anti‐tumour drugs cisplatin and fluorouracil, as evidenced by the decrease in blood urea nitrogen (BUN) (not serum creatinine [Scr]) and delayed onset of renal function failure, compared to that in non‐users.^12^ However, given the small size and the observational nature of this data, further investigation of co‐treatment of PPIs and cisplatin‐associated renal outcomes is needed.

A recent in vitro study focused on the mechanisms of PPI‐induced nephrotoxicity, pointing out that tubular cell death may be a contributing factor.^13^ Tubular cell death and tissue inflammation are major factors that determine the outcome of cisplatin‐induced AKI.^14^ Morphology analyses of cisplatin‐induced AKI have shown that tubular cells mainly undergo necrosis and apoptosis.^14^ We were curious to learn whether PPIs affect cisplatin‐induced renal damage via cell death and inflammation. To test this hypothesis, we assessed apoptosis, necroptosis and inflammation using lansoprazole, a commonly used PPI, in a cisplatin‐induced AKI model.

## 2 MATERIALS AND METHODS

### 2.1 Animal care and use

Male C57BL/6 mice (9‐10 weeks old; weight: 23‐25 g) were purchased from Guangdong Medical Laboratory Animal Center (Foshan, China, Quality certificate No. 44007200050890). The animals were housed under standardized pathogen‐free conditions (22‐24°C) under a 12 hours light/dark cycle at the Animal Center of Guangdong Medical University, with freely available water and food. Mice were randomly assigned to different groups: control group (CON, n = 5), lansoprazole group (LPZ, n = 6), cisplatin group (CIS, n = 6), cisplatin + lansoprazole 12.5 group (CIS + LPZ 12.5, n = 6) and cisplatin + lansoprazole 25 group (CIS + LPZ 25, n = 6). Cisplatin (S1166, Selleck) was administered via intraperitoneal (i.p.) injection at a single dose of 18 mg/kg. Lansoprazole (L8533, Sigma) was administered via i.p. injection at a dose of 12.5 mg/kg or 25 mg/kg 2 hours before cisplatin administration and then injected once daily for another 2 days. A dose of 25 mg/kg was used in the LPZ group. All mice were euthanized 3 days after cisplatin injection. All animal experiments were carried out with the approval of the Animal Experimentation Ethics Committee of Guangdong Medical University. All animal procedures were performed in strict accordance with the NIH Guide for the Care and Use of Laboratory Animals.

### 2.2 Measurement of kidney function

Blood samples were collected for kidney function measurement. Scr and BUN were tested according to the manufacturer's instructions (C011‐2‐1 and C013‐2, Nanjing Jiancheng Bioengineering Institute).

### 2.3 Histological evaluation of kidney damage

Kidney tissue was fixed with Carnoy's solution for 24 hours and embedded in paraffin as previously described.^15^ Renal morphology changes were examined on renal tissue slices (3 μm) by periodic acid–Schiff (PAS) staining and haematoxylin and eosin (HE) staining. Tubular dilatation, tubular cell necrosis and cast formation were scored from 0 to 4 as a percentage of the whole cortical area of the kidney slices.^16^ 0, normal; 1, <10%; 2, 10%‐25%; 3, 26%‐75%; 4, >75%. The morphometric measurements were performed in a blinded manner.

### 2.4 Quantitative real‐time PCR (RT‐qPCR)

Total RNA was extracted from kidney tissues using RNAiso Plus (Takara Bio Inc). PrimeScript RT Master Mix (Takara Bio Inc) was used to reverse transcribe the total RNA into cDNA according to the manufacturer's instructions. RT‐qPCR was performed using SYBR Premix Ex Taq II (Takara Bio Inc) with a Light Cycler 480 system (Roche). Sequences of the primers used were as follows: mouse *β‐actin* (forward) 5′‐GGCCAACCGTGAAAAGATGA‐3′ and (reverse) 5′‐ GACCAGAGGCATACAGGG ACA‐3′; mouse *Il‐1β* (forward) 5′‐CTTCAGGCAGGCAGTATCACTCAT‐3′ and (reverse) 5′‐TCTAATG GGAACGTCACACACCAG‐3′; mouse *Tnf‐α* (forward) 5′‐CATGAGCACAGAA AG CATGATCCG‐3′ and (reverse) 5′‐AAGCAGGAATGAGAAGAGGCTGAG‐3′; mouse *Nlrp3* (forward) 5′‐CTTCTCTGATGAGGCCCAAG‐3′ and (reverse) 5′‐GCAGCAAACTGGAAAGGAAG‐3′; mouse *Bcl‐2* (forward) 5′‐CTGGTGGA CAACATCGCTCTG‐3′ and (reverse) 5′‐GGTCTGCTGACCTCACTTGTG‐3′; mouse *Bax* (forward) 5′‐GCAAAGTAGAAGGCAACG‐3′ and (reverse) 5′‐GGCCAAGATCATCCATGACAACT‐3′; and mouse *Caspase 1* (forward) 5′‐ATGGCC GACAAGGTCCTG‐3′ and 5′‐TTAATGTCCTGGGAAGAGGTA‐3′. Relative gene expression was calculated using the 2^−ΔΔ^ *^Ct^* method, with *β‐actin* mRNA as a loading control. All mRNA primers were purchased from Sangon Biotech.

### 2.5 Western blotting analysis

Western blotting was performed as previously described.^17^ Briefly, kidney tissue or cells were lysed with the addition of a protease inhibitor cocktail (ST506‐2, Beyotime Institute of Biotechnology) and phosphatase inhibitor (P1260, Applygen) in radioimmunoprecipitation assay buffer and then centrifuged at 13 200 × *g* for 25 minutes at 4°C. Supernatants were extracted and denatured at 100°C for 15 minutes. After normalization, equal amounts of protein were loaded in each lane and separated by sodium dodecyl sulphate polyacrylamide gel electrophoresis using 10% or 12% gels, followed by transfer to polyvinylidene difluoride membranes (Millipore). The membranes were blocked in 5% (w/v) bovine serum albumin and incubated overnight at 4°C with the following primary antibodies: NLRP3 (ab6709, Multi Sciences), Bax (2772s, Cell Signaling Technology), Bcl‐2 (ab196495, Abcam), phosphorylated mixed lineage kinase domain‐like (p‐MLKL; ab196436, Abcam), receptor‐interacting protein kinase (RIPK) 3 (ab205421, Abcam), RIPK1 (17519‐1‐AP, Proteintech) and β‐actin (ab8227, Abcam). Subsequently, membranes were incubated with the corresponding horseradish peroxidase‐conjugated secondary antibodies (A0181, A0216 and A0208, Beyotime Institute of Biotechnology) for 1 hour. Protein bands were detected using Clarity Western ECL Substrate (Bio‐Rad) and imaged on the c500 Western Blot Imaging System (Azure Biosystems, Dublin, CA). Image J software (NIH, Bethesda, MD) was used for densitometric analysis.

### 2.6 Apoptosis detection

Apoptosis was determined by TUNEL staining (ApopTag Peroxidase Kit, EMD Millipore) in paraffin‐embedded renal tissues (3 μm), according to the manufacturer's instructions. The TUNEL‐positive cells in 10 high‐power fields at 400× magnification were counted.

### 2.7 Immunofluorescence

For detection of neutrophils, frozen kidney sections were incubated with primary antibody against Ly6G (BP0075‐1, BioXcell) overnight at 4°C, and then incubated with Alexa Fluor^®^ 594 goat anti‐rat IgG (A11005, Invitrogen) for 1 hour at room temperature in the dark. DAPI (C1005, Beyotime Biotechnology) was used for nuclear staining. Images were acquired using a fluorescence microscope (Olympus IX81, Olympus), and 10 high‐power fields at 400× magnification were randomly chosen for neutrophil number counting.

### 2.8 Electron microscopy

Ultrastructure analysis of renal proximal tubule cells was performed by transmission electron microscopy. Mouse renal tissue samples were fixed, dehydrated, embedded and stained as previously described.^17^ Ultrathin sections were examined using a JEM‐1400 transmission electron microscope (Jeol Ltd.).

### 2.9 Statistical analysis

Results are presented as the mean ± standard error. All statistical analyses were performed using GraphPad Prism version 5.0 (GraphPad Software, Inc). One‐way analysis of variance with Tukey's or Bonferroni's post‐hoc test was used to compare parameters among groups. Statistical significance was defined as *P* < .05.

## 3 RESULTS

### 3.1 Lansoprazole aggravates cisplatin‐induced AKI

**FIGURE 1:**
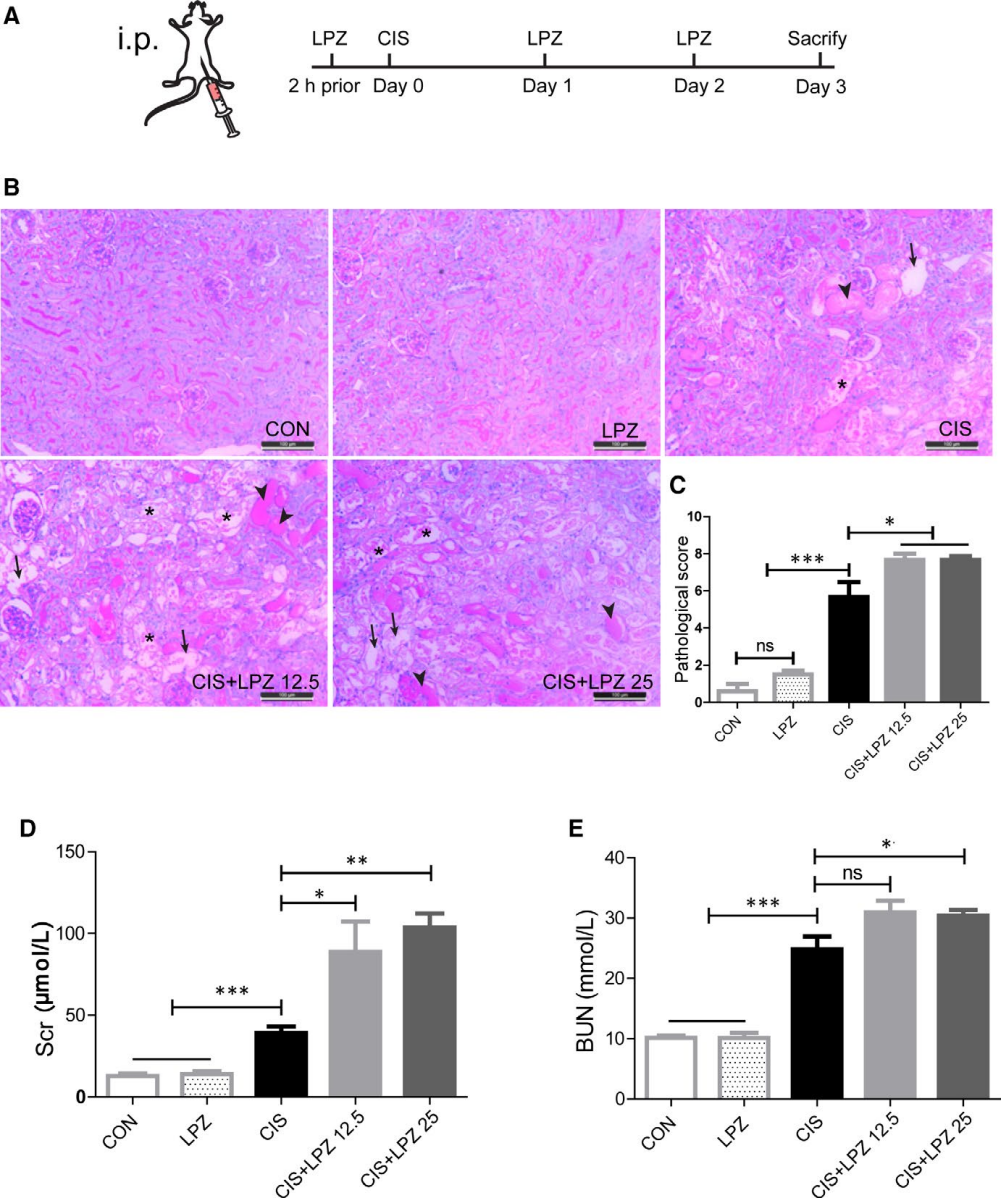
Lansoprazole aggravates acute kidney injury caused by cisplatin. C57BL/6 mice were injected with 18 mg/kg cisplatin or a combination with different doses of lansoprazole (LPZ). (A) Schematic representation of the experimental design. (B) Representative histology of renal tissue (periodic acid–Schiff [PAS] staining, ×200). Arrows indicate tubular dilatation. Arrowheads indicate tubular cast formation. Asterisks indicate tubular cell necrosis. (C) Pathological score of tubular injury in the kidneys. (D and E) Blood samples were collected to measure the concentration of serum creatinine (Scr) and blood urea nitrogen (BUN). Data are presented as mean ± standard error (SE). All data of each group are analyzed using one‐way analysis of variance (ANOVA). ns: not significant; *P < .05; **P < .01; and ***P < .001

We used the PPI, lansoprazole, to investigate the effect of PPIs on cisplatin‐induced AKI. An estimated human clinical equivalent dose (or range of doses) of lansoprazole (12.5 mg/kg) and a higher dose (25 mg/kg) were tested in this experiment. Based on the doses used in previous experiments,^18^ a single dose of 18 mg/kg cisplatin was chosen to induce AKI in C57BL/6 mice. The experimental design is illustrated in Figure 1A. According to PAS staining, there were no significant differences in morphology between the LPZ and CON groups (Figure 1B,C). Similar to the results of previous studies,^19^ tubular dilatation, tubular cell necrosis and cast formation were observed in renal sections following cisplatin treatment. When co‐treated with different doses of lansoprazole in addition to cisplatin, renal damage significantly increased compared to that in cisplatin treatment alone (Figure 1B,C). This result was further confirmed by the remarkable upregulation of Scr and BUN in the CIS + LPZ 12.5 and CIS + LPZ 25 groups compared to that in the CIS group (Figure 1D,E), indicating the exacerbation of renal function caused by lansoprazole. Taken together, these data show that lansoprazole aggravates cisplatin‐induced AKI.

### 3.2 Exacerbation of cisplatin‐induced AKI by lansoprazole is independent of tubular apoptosis

**FIGURE 2:**
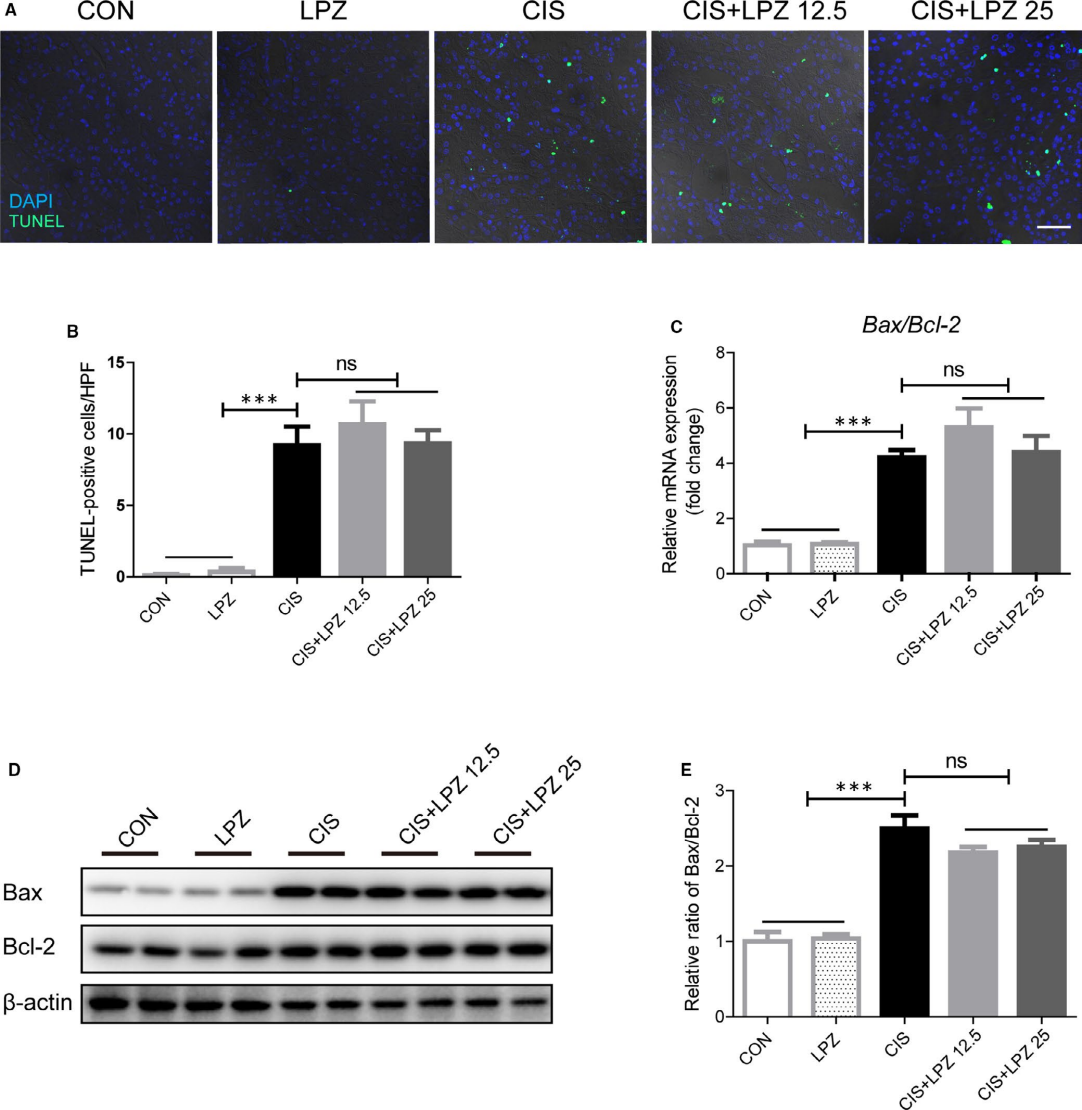
Exacerbation of cisplatin‐induced kidney injury by lansoprazole is independent of tubular apoptosis. (A) Representative images of TUNEL staining (×400). (B) Quantification of TUNEL‐positive cells in different groups. (C) Relative mRNA level of *Bax/Bcl‐2*. (D) Detection of Bax/Bcl‐2 protein level in renal tissues via western blot. (E) Quantification of Bax/Bcl‐2 protein level expressed as mean ± standard error (SE). All data of each group are analysed using one‐way analysis of variance (ANOVA). ns: not significant; ***P < .001

A recent study revealed that PPIs nephrotoxicity is associated with tubular cell death.^13^ Since tubular apoptosis is one of the major cellular process changes in cisplatin‐induced nephrotoxicity,^20^ we questioned whether lansoprazole exacerbates cisplatin‐induced renal damage via apoptosis. The TUNEL assay showed that cisplatin treatment led to significant tubular cell apoptosis, while no further increase in apoptosis was observed after co‐treatment with different doses of lansoprazole (Figure 2A,B). Moreover, the critical apoptosis regulators Bax (pro‐apoptotic) and Bcl‐2 (anti‐apoptotic) were tested at both the mRNA and protein levels. The Bax/Bcl‐2 ratio has been hypothesized to increase apoptosis.^21^ A remarkable increase in the Bax/Bcl‐2 ratio at both the mRNA and protein levels indicated apoptosis in renal tissue after cisplatin treatment. No further increase in the Bax/Bcl‐2 ratio was detected following addition of different doses of lansoprazole (Figure 2C‐E). Taken together, these data suggest that apoptosis may not explain why lansoprazole aggravates cisplatin‐induced AKI.

### 3.3 Lansoprazole increases cisplatin‐induced tubular necroptosis

**FIGURE 3:**
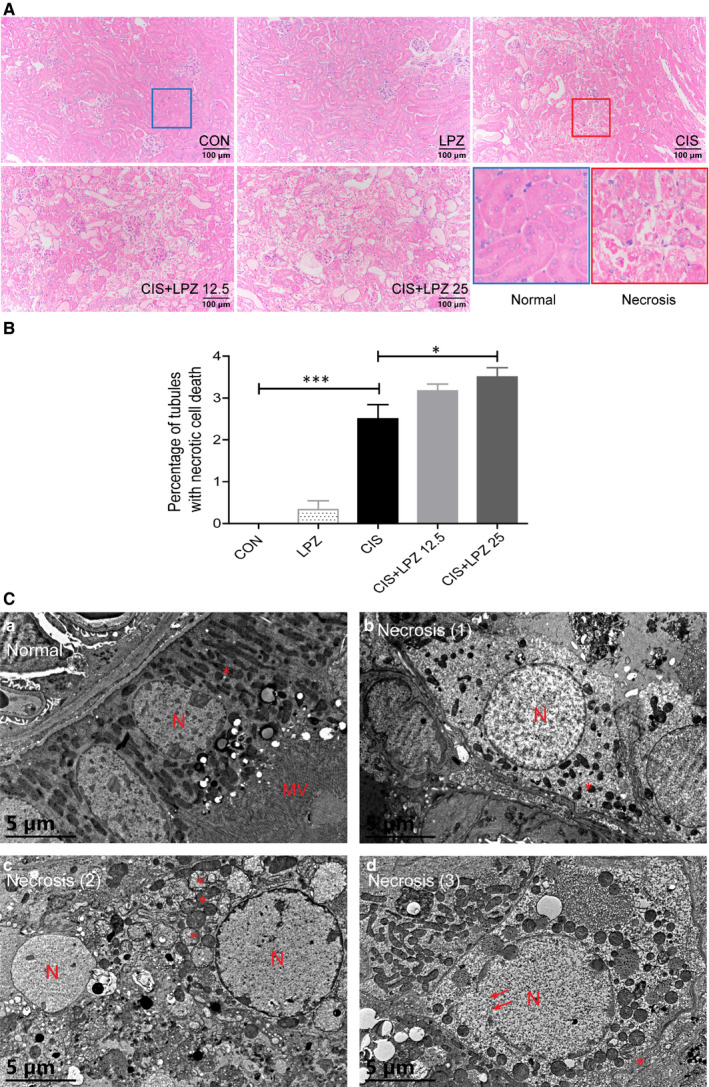
Lansoprazole increases cisplatin‐induced tubular necrosis. (A) Representative histology of haematoxylin and eosin (HE) stained renal tissue (×200). (B) Quantifications of the percentage of necrotic proximal tubules in HE‐stained renal tissue. (C) Representative electron micrographs of normal or necrotic proximal renal tubule cells from control mice and mice receiving cisplatin and/or lansoprazole (original magnification, ×8000). (a) Normal proximal renal tubule cells showing rounded nucleus (N), abundant mitochondria (asterisk) and prominent microvilli (MV); necrotic epithelial cells (b‐d) showing pale, swollen and ruptured nuclei (d, arrows), damage and loss of mitochondria, and necrotic cellular debris with hardly recognizable structures (c, arrowheads). Results are expressed as mean ± standard error (SE). All data of each group are analysed using one‐way analysis of variance (ANOVA). *P < .05

**FIGURE 4:**
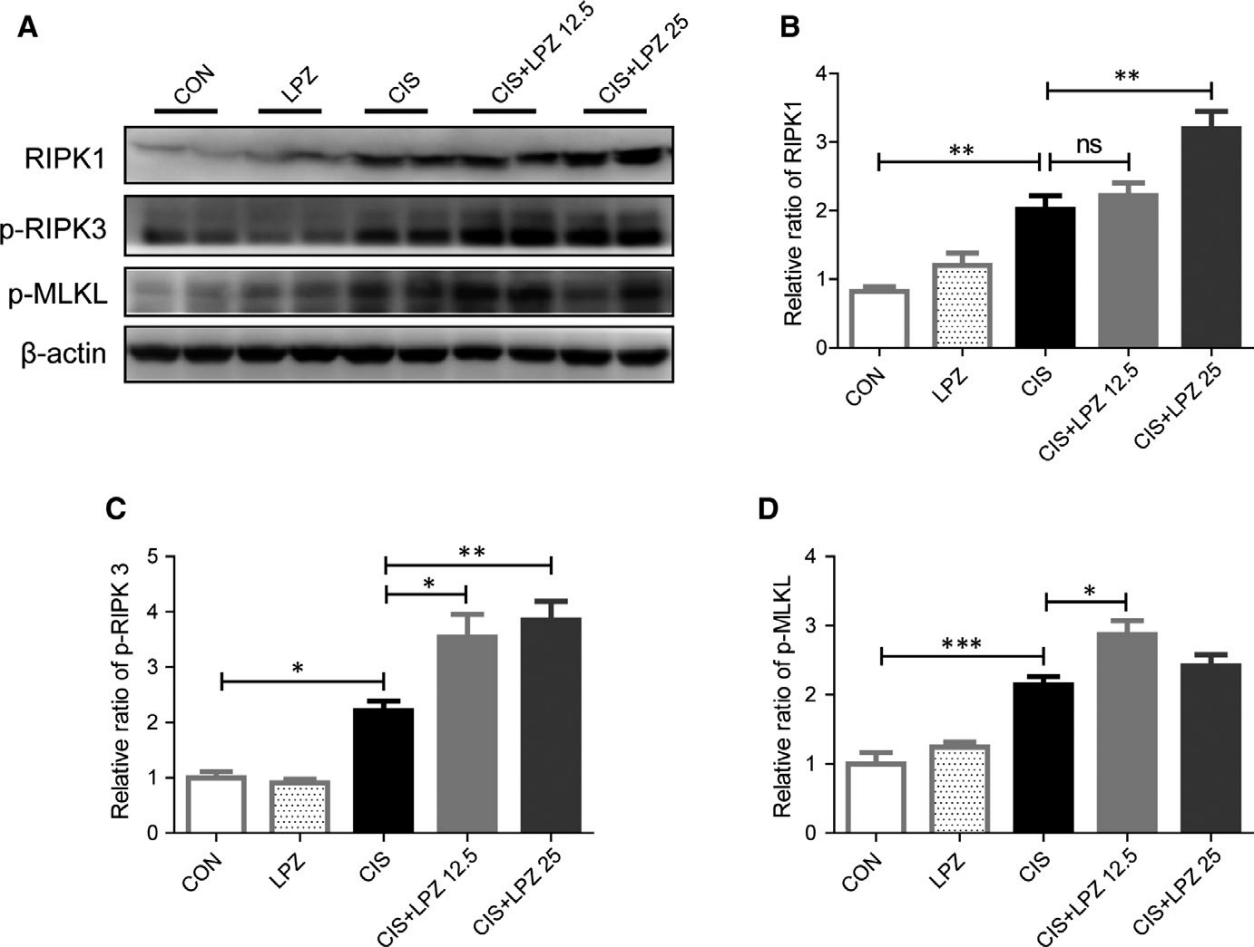
Necroptosis may contribute to the exacerbation of cisplatin‐induced kidney injury by lansoprazole. (A) Necroptosis associated proteins, p‐MLKL, p‐RIPK3 and RIPK1, determined by western blot. (B‐D) Quantifications of p‐MLKL, p‐RIPK3 and RIPK1 protein levels expressed as mean ± standard error (SE). All data of each group are analysed using one‐way analysis of variance (ANOVA). ns: not significant; *P < .05; **P < .01; and ***P < .001

Next, we estimated the occurrence of necroptosis, another common form of tubular cell death in cisplatin‐induced renal injury.^19^ Necroptosis, a type of programmed necrosis, can be triggered by toll‐like receptors, interferons, death receptors and other mediators.^22^ RIPK3 and its substrate MLKL are considered crucial players of this pathway.^23^ Here, we investigated the role of necroptosis in the exacerbation of cisplatin‐induced kidney injury by lansoprazole. Tubular necrosis was estimated in HE‐stained renal tissues (Figure 3A). Cisplatin significantly induced necrosis in tubular cells. The percentage of necrotic tubular cells increased slightly after 12.5 mg/kg lansoprazole combination treatment and significantly increased following treatment with 25 mg/kg lansoprazole, compared to that after cisplatin treatment alone (Figure 3B). Next, ultrastructural changes in renal proximal tubule cells were detected under normal conditions or after cisplatin treatment with or without lansoprazole combination. Representative electron micrographs of necrotic tubular cells showed swollen and ruptured nuclei and loss of cell organelle content and integrity (Figure 3C). Subsequently, we examined necroptotic biomarkers, including RIPK1, p‐RIPK3 and p‐MLKL. We detected necroptosis in the renal tissue after cisplatin treatment, as evidenced by the significant increase in RIPK1, p‐RIPK3 and p‐MLKL expression (Figure 4). Both p‐MLKL and p‐RIPK3 expression showed a further significant increase in the CIS + LPZ groups compared to that in the CIS group. RIPK1 expression also showed a slight increase in the CIS + LPZ 12.5 group but showed a significant increase in the CIS + LPZ 25 group compared to that in the CIS group.

In in vitro study, cisplatin were used to stimulate HK‐2 cells for 24 hours at 80 μmol/L.^19^ Different doses of LPZ, 2.5, 5, 10 and 50 μmol/L, were co‐treated with cisplatin, among which the concentration of 5 μmol/L is within the therapeutic range in clinic treatment.^24^ Consistently, increased cell death was observed in CIS + LPZ groups, compared with CIS group, revealed by propidium iodide (PI) stain with flow cytometry (Figure S1). PARP‐1, RIPK1, p‐RIPK3 and p‐MLKL expression were also tested by western blot. The core necroptosis execution component, p‐MLKL, was significantly increased after co‐treated with LPZ, compared with cisplatin administration alone (Figure S2). Altogether, these results demonstrate that LPZ aggravates cisplatin‐induced tubular necroptosis.

### 3.4 Lansoprazole enhances cisplatin‐induced renal inflammation

**FIGURE 5:**
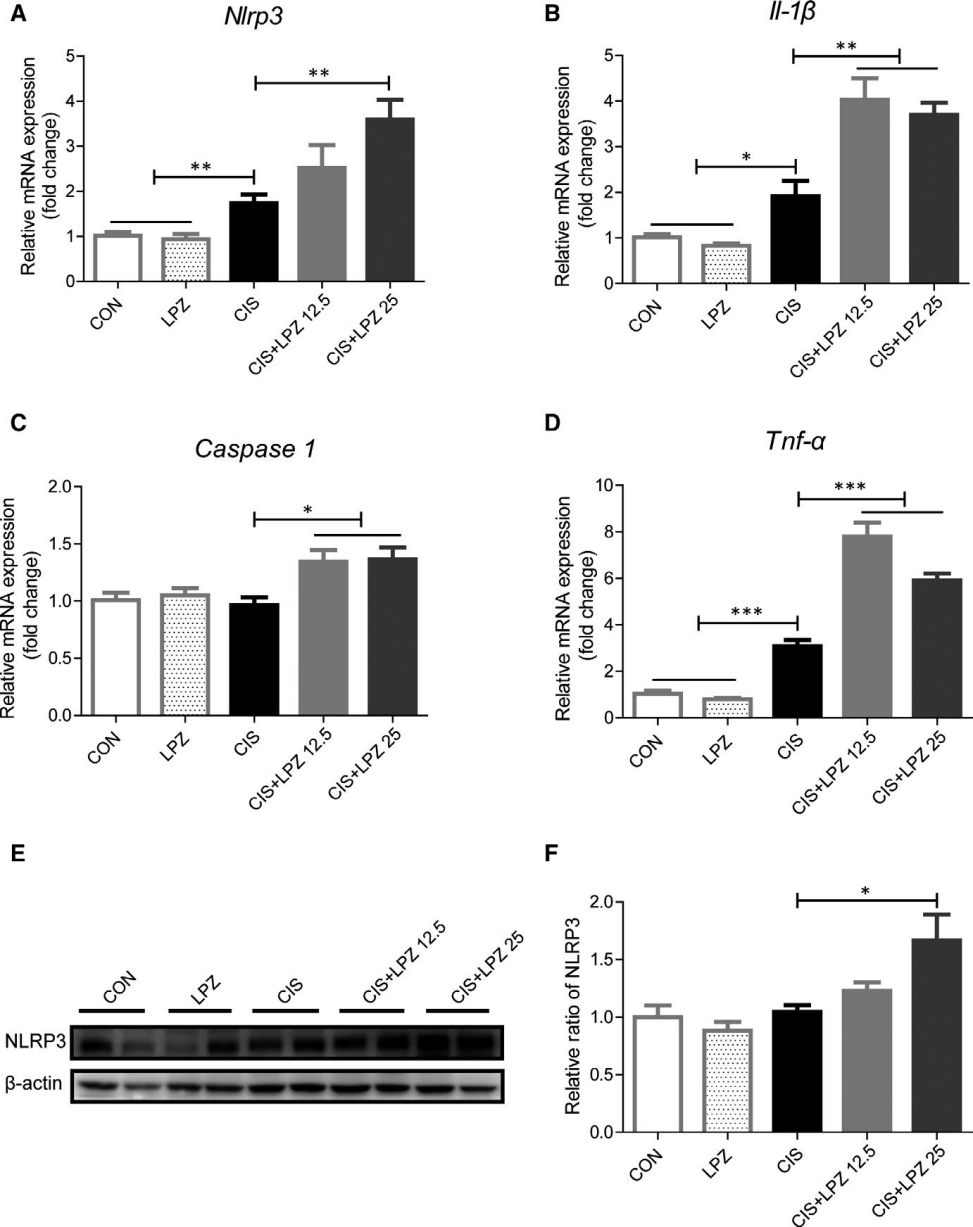
Lansoprazole enhances the expression of pro‐inflammation cytokines. (A‐D) mRNA expression of inflammatory proteins in the kidneys of different groups, such as *Nlrp3*, *Il‐1β*, *Caspase 1* and *Tnf‐α*, measured by RT‐qPCR. (E) NLRP3 protein detected by western blot in renal tissue. (F) Quantitative level of NLRP3 protein expression. Results are expressed as mean ± standard error (SE). All data of each group are analysed using one‐way analysis of variance (ANOVA). *P < .05; **P < .01; and ***P < .001

**FIGURE 6:**
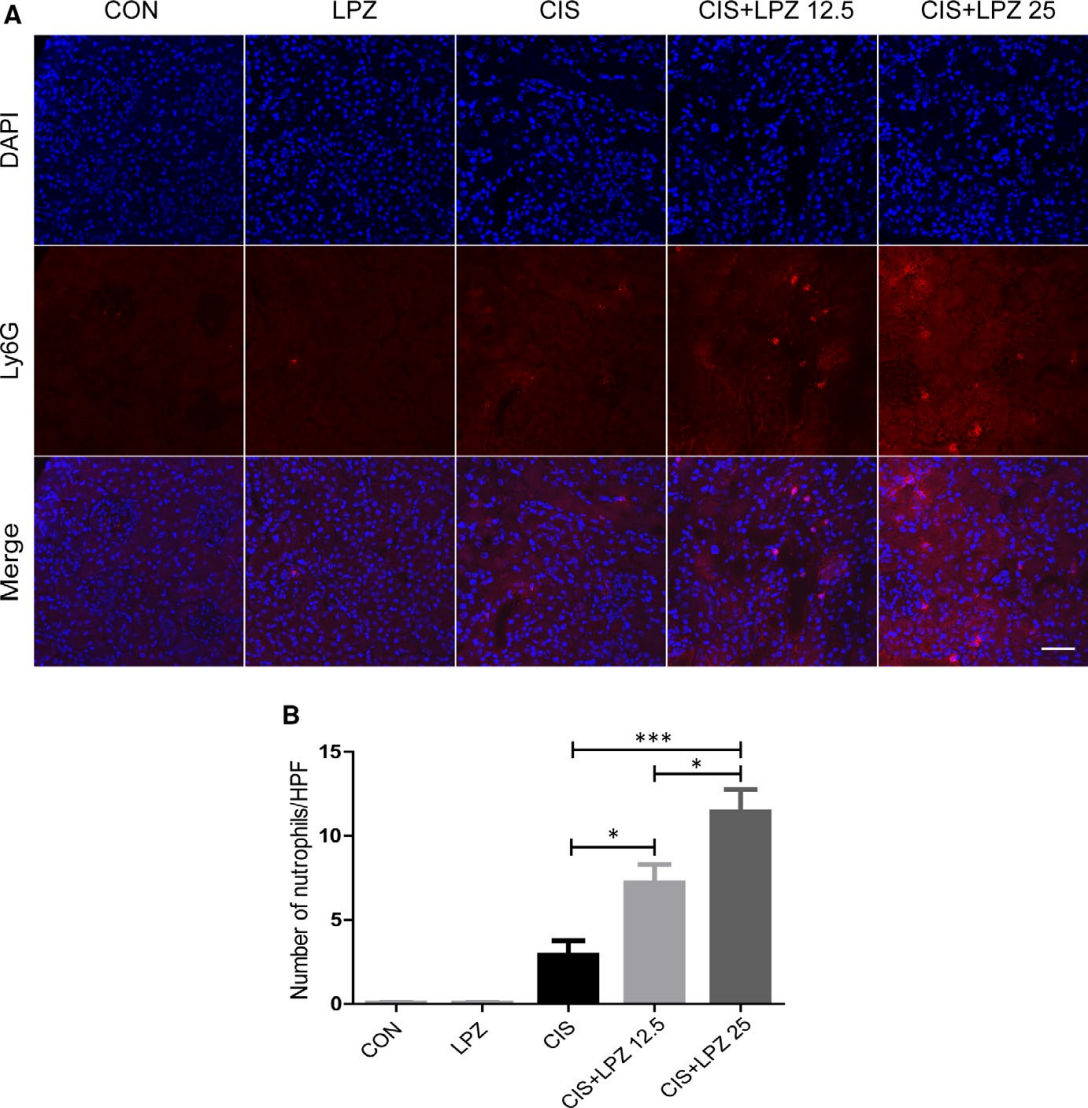
Lansoprazole enhances neutrophil infiltration. (A) Neutrophil infiltration in renal tissue determined via Ly6G immunofluorescent labelling (×400). (B) Quantification of Ly6G immunofluorescence staining. Results are expressed as mean ± standard error (SE). All data of each group are analysed using one‐way analysis of variance (ANOVA). *P < .05

In vivo data have shown that necroptosis plays a critical role in triggering inflammation.^25^ Next, we were keen to determine whether lansoprazole enhances cisplatin‐induced renal inflammation. We observed that cisplatin treatment significantly increased the mRNA expression of some pro‐inflammatory factors, such as NLRP3, TNF‐α and IL‐1β, but not caspase 1, compared to that in the control and lansoprazole treatments. Notably, the combination of different doses of lansoprazole and cisplatin further increased the mRNA expression of these pro‐inflammatory cytokines compared to that in cisplatin treatment alone (Figure 5A‐C). NLRP3, caspase 1 and IL‐1β were also tested at the protein level, and it showed a slight increase in the CIS + LPZ 12.5 group and a significant increase in the CIS + LPZ 25 group compared to that in the CIS group (Figure 5D,E and Figure S3). Besides, we also test IL‐6 and IL‐18 by western blot in in vitro study. The result shows that both IL‐6 and IL‐18 increased in CIS + LPZ groups, compared with CIS group (Figure S4). In addition, we tested neutrophil infiltration in kidney tissues, and the result was consistent with the expression of pro‐inflammatory factors (Figure 6A,B). Collectively, these data suggest that lansoprazole increases cisplatin‐induced renal inflammation.

## 4 DISCUSSION

**FIGURE 7:**
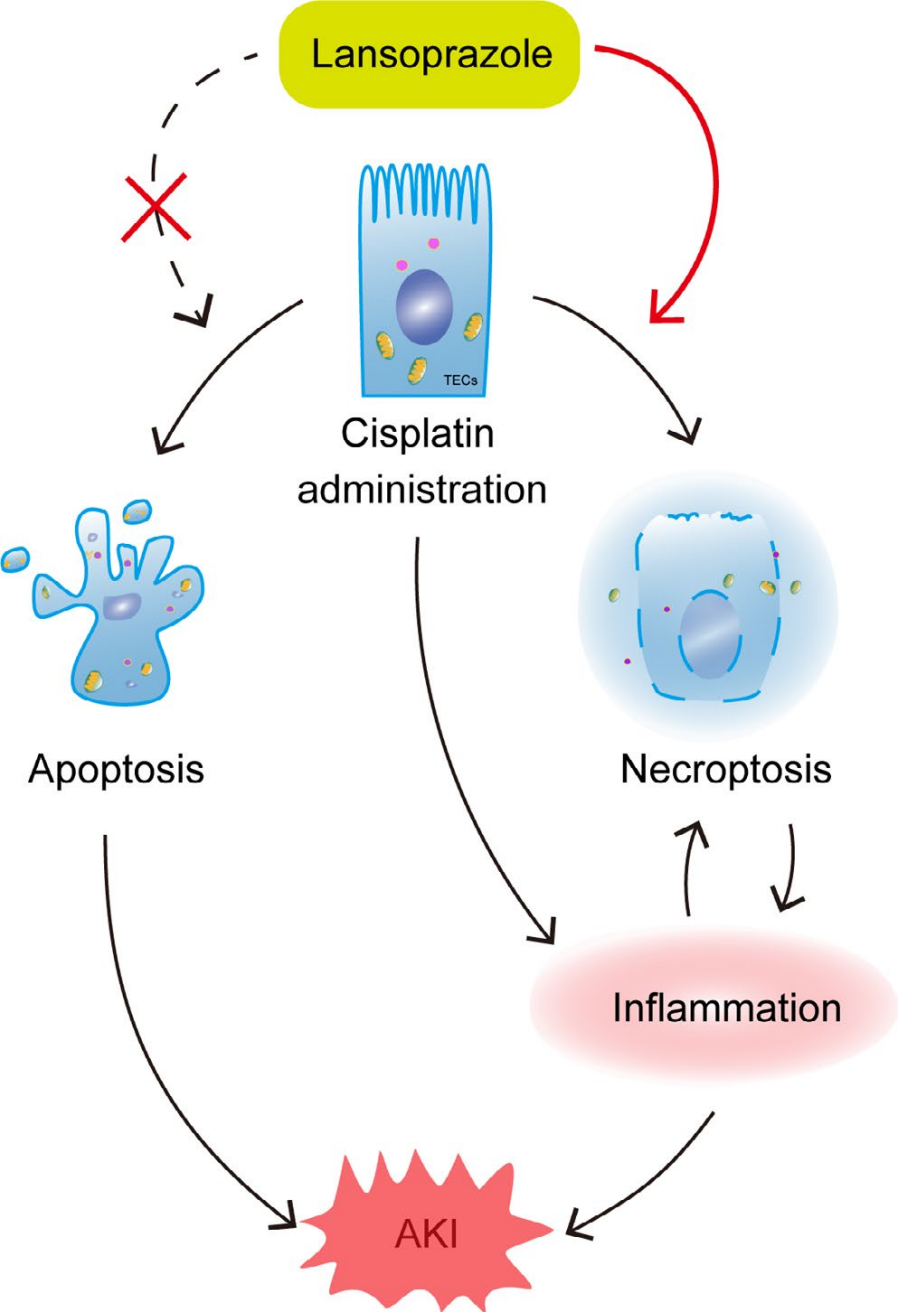
Current working hypothesis. Lansoprazole aggravates cisplatin‐induced acute kidney injury. This is associated with increased necroptosis in tubular epithelial cells, but not apoptosis, leading to an increased inflammatory reaction

In the present study, we examined the effect of lansoprazole, a commonly used PPI, on kidney injury induced by cisplatin treatment. Our data demonstrate that lansoprazole aggravates cisplatin‐induced AKI. Necroptosis and inflammation may contribute to this aggravated renal injury, but this exacerbation is independent of apoptosis (Figure 7).

In this study, a dose of 12.5 mg/kg or 25 mg/kg lansoprazole was used according to the conversion of the clinical dosage (lansoprazole, 1 or 2 mg/kg/d). The pattern of lansoprazole treatment was also based on clinical practice in cisplatin‐treated patients. Our findings showed that lansoprazole aggravated cisplatin‐induced AKI. However, in another similar study, rats were pretreated with PPIs (omeprazole, 1.8 mg/kg and 3.6 mg/kg) before cisplatin treatment, in which the doses of omeprazole were below the range of clinical treatment and showed a renoprotective effect.^26^ The inconsistencies in these outcomes may be owing to the different doses and administration pattern of PPIs.

Recently, an in vitro study revealed that tubular cell death may contribute to omeprazole nephrotoxicity.^13^ Therefore, we investigated the role of renal tubular cell death in exacerbated renal function after cisplatin and lansoprazole co‐treatment. Renal tubular cell death (apoptosis and necrosis) has been hypothesized as a major factor in cisplatin‐induced nephrotoxicity.^27^ Over the past two decades, apoptosis in tubular epithelial cells has been well studied and has been considered to be a promising therapeutic target.^28^, ^29^ In the present study, our data show an increased Bax/Bcl‐2 ratio at both the mRNA and protein level and TUNEL staining after cisplatin treatment, demonstrating that cisplatin does indeed induce apoptosis. However, no further increase in apoptosis was observed following combination treatment with lansoprazole and cisplatin, indicating that apoptosis may not contribute to the exacerbated renal function. In fact, the therapeutic role of apoptosis in cisplatin‐induced renal dysfunction has been argued. A recent study showed that the prevention of proximal renal tubular apoptosis did not rescue cisplatin‐induced kidney dysfunction.^30^ Furthermore, a broadly used antiapoptotic agent zVAD‐fmk has been reported to worsen cisplatin‐induced decline of renal function by impairing autophagic flux.^31^ Our results of morphology analyses showed obvious necrosis in tubular cells after cisplatin treatment, which is consistent with the results of a previous study showing that necrosis is the main form of cell death in cisplatin‐induced nephrotoxicity, rather than apoptosis.^14^ Notably, lansoprazole significantly increased the number of necrotic tubules, which may account for the deteriorative renal function. Necrosis has long been thought to be an uncontrolled form of cell death. In recent years, significant progress has aided our understanding of necroptosis as a type of programmed necrosis. It has also been reported that cisplatin induces necroptosis via the TNF‐α‐mediated RIPK1/RIPK3/MLKL pathway.^32^ In addition, necroptosis has been confirmed to be the major proximal tubular cell death pathway in cisplatin‐induced AKI by manipulating the *RIPK3* or *MLKL* gene in vivo.^19^ RIPK1 can assemble RIPK3 to form a RIPK1‐RIPK3 hetero‐interaction, which can promote RIPK3‐RIPK3 homo ‐interactions, resulting in the recruitment and phosphorylation of MLKL.^33^, ^34^ As an executor, p‐MLKL functions as either a platform for Na^+^ or Ca^++^ channel recruitment or a pore in the plasma membrane, causing ion influx and loss of plasma membrane integrity, respectively.^35^ Accordingly, we hypothesized that necroptosis contributed to the worsening of renal function. We found that RIPK1, p‐RIPK3 and p‐MLKL showed a significant increase after cisplatin treatment and a further increase in protein expression after co‐treatment with lansoprazole and cisplatin both in vivo and in vitro, suggesting that necroptosis is involved in the exacerbation of cisplatin‐induced renal injury.

Several studies have shown that necroptosis is involved in promoting inflammation.^36^, ^37^ A recent study showed that inflammatory cytokine upregulation, such as the TNF‐α family of cytokines, was partially declined in *RIPK3*‐ or *MLKL*‐deficient cisplatin‐treated mice,^19^, ^23^ indicating necroptosis take part in forming a inflammation state. Also, TNF, Fas and TRAIL, as well as certain TLR ligand can induce necroptosis,^38^ forming a positive feedback loop between inflammation and necroptosis.^19^ In our study, the increased inflammatory cytokine transcription reflects a more severe inflammatory condition in the CIS + LPZ groups compared to that in the CIS group both in in vivo and in vitro study. Moreover, the increased infiltration of neutrophils further confirmed the enhanced inflammation after co‐treatment with lansoprazole and cisplatin. Accordingly, necroptosis and inflammation have their distinct functions or may form a positive feedback pattern to cause the aggravated renal damage in combination with lansoprazole and cisplatin.

Our study has some limitations. First, necroptosis‐related gene knockdown experiments should be performed in vivo to estimate the role of PPIs in the aggravation of cisplatin‐induced renal injury. Furthermore, additional efforts should be made to elucidate the precise mechanism by which PPIs enhance necroptosis in the setting of cisplatin‐induced renal injury. Although, some efforts that we have made give us some hints that this may be associated with increased production of mitochondrial ROS (Figure S5). Our findings also need to be proven in human studies.

Taken together, our results suggest that lansoprazole aggravates AKI induced by cisplatin and that necroptosis may be involved in the exacerbation of this kidney damage.

## CONFLICT OF INTEREST

All authors declared no conflict of interests.

## AUTHOR CONTRIBUTIONS

**Lin Ye:** Data curation (lead); Formal analysis (equal); Investigation (lead); Methodology (equal); Project administration (equal); Software (equal); Supervision (equal); Validation (equal); Visualization (equal); Writing‐original draft (equal); Writing‐review & editing (equal). **Wanxia Pang:** Data curation (equal); Formal analysis (equal); Investigation (equal); Methodology (equal); Resources (equal); Validation (equal); Writing‐original draft (supporting); Writing‐review & editing (equal). **Yanheng Huang:** Formal analysis (equal); Investigation (equal); Methodology (equal); Software (equal); Validation (equal); Visualization (equal); Writing‐review & editing (equal). **Hongluan Wu:** Investigation (equal); Methodology (equal); Software (equal); Validation (equal); Visualization (equal). **Xiaorong Huang:** Formal analysis (supporting); Investigation (equal); Methodology (supporting); Visualization (supporting); Writing‐review & editing (supporting). **Jianxing Liu:** Investigation (supporting); Methodology (equal); Validation (equal); Writing‐review & editing (supporting). **Shujun Wang:** Funding acquisition (equal); Resources (equal); Validation (equal); Writing‐review & editing (equal). **Chen Yang:** Conceptualization (equal); Funding acquisition (equal); Methodology (supporting); Project administration (supporting); Resources (equal); Writing‐review & editing (supporting). **Qingjun Pan:** Data curation (supporting); Project administration (equal); Supervision (equal); Validation (equal); Writing‐review & editing (equal). **Huafeng Liu:** Conceptualization (supporting); Data curation (equal); Funding acquisition (lead); Project administration (equal); Resources (lead); Supervision (equal); Validation (equal); Writing‐review & editing (supporting).

## Supporting information

Figure S1

Figure S2

Figure S3

Figure S4

Figure S5

Supporting material

## Data Availability

All data included in this study are available upon request by contact with the corresponding author.

## References

[1] Hamroun A , Lenain R , Bigna JJ , et al. Prevention of cisplatin‐induced acute kidney injury: a systematic review and meta‐analysis. Drugs. 2019;79(14):1567‐1582. 10.1007/s40265-019-01182-1 31429065

[2] Xu X , Nie S , Liu Z , et al. Epidemiology and clinical correlates of AKI in Chinese hospitalized adults. Clin J Am Soc Nephrol. 2015;10(9):1510‐1518. 10.2215/CJN.02140215 26231194 PMC4559507

[3] Crona DJ , Faso A , Nishijima TF , McGraw KA , Galsky MD , Milowsky MI . A systematic review of strategies to prevent cisplatin‐induced nephrotoxicity. Oncologist. 2017;22(5):609‐619. 10.1634/theoncologist.2016-0319 28438887 PMC5423518

[4] Casanova AG , Hernández‐Sánchez MT , López‐Hernández FJ , et al. Systematic review and meta‐analysis of the efficacy of clinically tested protectants of cisplatin nephrotoxicity. Eur J Clin Pharmacol. 2020;76(1):23‐33. 10.1007/s00228-019-02771-5 31677116

[5] Eriksson S , Långström G , Rikner L , Carlsson R , Naesdal J . Omeprazole and H2‐receptor antagonists in the acute treatment of duodenal ulcer, gastric ulcer and reflux oesophagitis: a meta‐analysis. Eur J Gastro Hepatol. 1995;7(5):467‐475. 7614110

[6] Smelick GS , Heffron TP , Chu L , et al. Prevalence of acid‐reducing agents (ARA) in cancer populations and ARA drug‐drug interaction potential for molecular targeted agents in clinical development. Mol Pharm. 2013;10(11):4055‐4062. 10.1021/mp400403s 24044612

[7] Xu X , Nie S , Zhang A , et al. Acute kidney injury among hospitalized children in China. Clin J Am Soc Nephrol. 2018;13(12):1791‐1800. 10.2215/cjn.00800118 30287424 PMC6302328

[8] Hart E , Dunn TE , Feuerstein S , Jacobs DM . Proton pump inhibitors and risk of acute and chronic kidney disease: a retrospective cohort study. Pharmacotherapy. 2019;39(4):443‐453. 10.1002/phar.2235 30779194 PMC6453745

[9] Lazarus B , Chen Y , Wilson FP , et al. Proton pump inhibitor use and the risk of chronic kidney disease. JAMA Intern Med. 2016;176(2):238‐246. 10.1001/jamainternmed.2015.7193 26752337 PMC4772730

[10] Xie Y , Bowe B , Li T , Xian H , Balasubramanian S , Al‐Aly Z . Proton pump inhibitors and risk of incident CKD and progression to ESRD. J Am Soc Nephrol. 2016;27(10):3153‐3163. 10.1681/asn.2015121377 27080976 PMC5042677

[11] Forgacs I , Loganayagam A . Overprescribing proton pump inhibitors. BMJ. 2008;336(7634):2‐3. 10.1136/bmj.39406.449456.BE PMC2174763 18174564

[12] Ikemura K , Oshima K , Enokiya T , et al. Co‐administration of proton pump inhibitors ameliorates nephrotoxicity in patients receiving chemotherapy with cisplatin and fluorouracil: a retrospective cohort study. Cancer Chemother Pharmacol. 2017;79(5):943‐949. 10.1007/s00280-017-3296-7 28364288

[13] Fontecha‐Barriuso M , Martín‐Sanchez D , Martinez‐Moreno JM , et al. Molecular pathways driving omeprazole nephrotoxicity. Redox Biol. 2020;32:101464. 10.1016/j.redox.2020.101464 32092686 PMC7038587

[14] Ozkok A , Edelstein CL . Pathophysiology of cisplatin‐induced acute kidney injury. Biomed Res Int. 2014;2014:1‐17. 10.1155/2014/967826 PMC4140112 25165721

[15] Yang C , Xue J , An N , et al. Accelerated glomerular cell senescence in experimental lupus nephritis. Med Sci Monit. 2018;24:6882‐6891. 10.12659/msm.909353 30265659 PMC6180956

[16] Pieters TT , Falke LL , Nguyen TQ , et al. Histological characteristics of Acute Tubular Injury during Delayed Graft Function predict renal function after renal transplantation. Physiol Rep. 2019;7(5):e14000. 10.14814/phy2.14000 30821122 PMC6395310

[17] Liu WJ , Xu BH , Ye L , et al. Urinary proteins induce lysosomal membrane permeabilization and lysosomal dysfunction in renal tubular epithelial cells. Am J Physiol Renal Physiol. 2015;308(6):F639‐F649. 10.1152/ajprenal.00383.2014 25587119

[18] Kim TW , Kim YJ , Kim HT , Park SR , Jung JY . β‐Lapachone enhances Mre11‐Rad50‐Nbs1 complex expression in cisplatin‐induced nephrotoxicity. Pharmacol Rep. 2016;68(1):27‐31. 10.1016/j.pharep.2015.06.007 26721347

[19] Xu Y , Ma H , Shao J , et al. A role for tubular necroptosis in cisplatin‐induced AKI. J Am Soc Nephrol. 2015;26(11):2647‐2658. 10.1681/asn.2014080741 25788533 PMC4625668

[20] Wei Q , Dong G , Franklin J , Dong Z . The pathological role of Bax in cisplatin nephrotoxicity. Kidney Int. 2007;72(1):53‐62. 10.1038/sj.ki.5002256 17410096

[21] Hoshyar R , Bathaie SZ , Sadeghizadeh M . Crocin triggers the apoptosis through increasing the Bax/Bcl‐2 ratio and caspase activation in human gastric adenocarcinoma, AGS, cells. DNA Cell Biol. 2013;32(2):50‐57. 10.1089/dna.2012.1866 23347444

[22] Dhuriya YK , Sharma D . Necroptosis: a regulated inflammatory mode of cell death. J Neuroinflammation. 2018;15(1):199. 10.1186/s12974-018-1235-0 29980212 PMC6035417

[23] Wang H , Sun L , Su L , et al. Mixed lineage kinase domain‐like protein MLKL causes necrotic membrane disruption upon phosphorylation by RIP3. Mol Cell. 2014;54(1):133‐146. 10.1016/j.molcel.2014.03.003 24703947

[24] Association MMCoCH . Pharmaceutical Care for the Clinical Application of Proton Pump Inhibitors (1st ed.). People's Medical Publishing House; 2013.

[25] Pasparakis M , Vandenabeele P . Necroptosis and its role in inflammation. Nature. 2015;517(7534):311‐320. 10.1038/nature14191 25592536

[26] Gao H , Zhang S , Hu T , et al. Omeprazole protects against cisplatin‐induced nephrotoxicity by alleviating oxidative stress, inflammation, and transporter‐mediated cisplatin accumulation in rats and HK‐2 cells. Chem Biol Interact. 2019;297:130‐140. 10.1016/j.cbi.2018.11.008 30452898

[27] Blachley JD , Hill JB . Renal and electrolyte disturbances associated with cisplatin. Ann Intern Med. 1981;95(5):628‐632. 10.7326/0003-4819-95-5-628 7027859

[28] Lieberthal W , Triaca V , Levine J . Mechanisms of death induced by cisplatin in proximal tubular epithelial cells: apoptosis vs. necrosis. Am J Physiol. 1996;270(4):F700‐F708. 10.1152/ajprenal.1996.270.4.F700 8967349

[29] Cummings BS , Schnellmann RG . Cisplatin‐induced renal cell apoptosis: caspase 3‐dependent and ‐independent pathways. J Pharmacol Exp Ther. 2002;302(1):8‐17. 10.1124/jpet.302.1.8 12065694

[30] Sridevi P , Nhiayi MK , Wang JY . Genetic disruption of Abl nuclear import reduces renal apoptosis in a mouse model of cisplatin‐induced nephrotoxicity. Cell Death Differ. 2013;20(7):953‐962. 10.1038/cdd.2013.42 23660976 PMC3679464

[31] Herzog C , Yang C , Holmes A , Kaushal GP . zVAD‐fmk prevents cisplatin‐induced cleavage of autophagy proteins but impairs autophagic flux and worsens renal function. Am J Physiol Renal Physiol. 2012;303(8):F1239‐F1250. 10.1152/ajprenal.00659.2011 22896037 PMC3469677

[32] Xu Y , Ma HB , Fang YL , et al. Cisplatin‐induced necroptosis in TNFα dependent and independent pathways. Cell Signal. 2017;31:112‐123. 10.1016/j.cellsig.2017.01.004 28065786

[33] Cho YS , Challa S , Moquin D , et al. Phosphorylation‐driven assembly of the RIP1‐RIP3 complex regulates programmed necrosis and virus‐induced inflammation. Cell. 2009;137(6):1112‐1123. 10.1016/j.cell.2009.05.037 19524513 PMC2727676

[34] Wu XN , Yang ZH , Wang XK , et al. Distinct roles of RIP1‐RIP3 hetero‐ and RIP3‐RIP3 homo‐interaction in mediating necroptosis. Cell Death Differ. 2014;21(11):1709‐1720. 10.1038/cdd.2014.77 24902902 PMC4211369

[35] Cai Z , Jitkaew S , Zhao J , et al. Plasma membrane translocation of trimerized MLKL protein is required for TNF‐induced necroptosis. Nat Cell Biol. 2014;16(1):55‐65. 10.1038/ncb2883 24316671 PMC8369836

[36] Lin J , Kumari S , Kim C , et al. RIPK1 counteracts ZBP1‐mediated necroptosis to inhibit inflammation. Nature. 2016;540(7631):124‐128. 10.1038/nature20558 27819681 PMC5755685

[37] Wang JN , Liu MM , Wang F , et al. RIPK1 inhibitor Cpd‐71 attenuates renal dysfunction in cisplatin‐treated mice via attenuating necroptosis, inflammation and oxidative stress. Clin Sci. 2019;133(14):1609‐1627. 10.1042/cs20190599 31315969

[38] Kearney CJ , Martin SJ . An inflammatory perspective on necroptosis. Mol Cell. 2017;65(6):965‐973. 10.1016/j.molcel.2017.02.024 28306512

