# Supplementary figures and images for: Lansoprazole promotes cisplatin‐induced acute kidney injury via enhancing tubular necroptosis
Source: J Cell Mol Med. 2021 Feb 18;25(5):2703–13. doi: 10.1111/jcmm.16302 (PMC7933939; doi:10.1111/jcmm.16302)

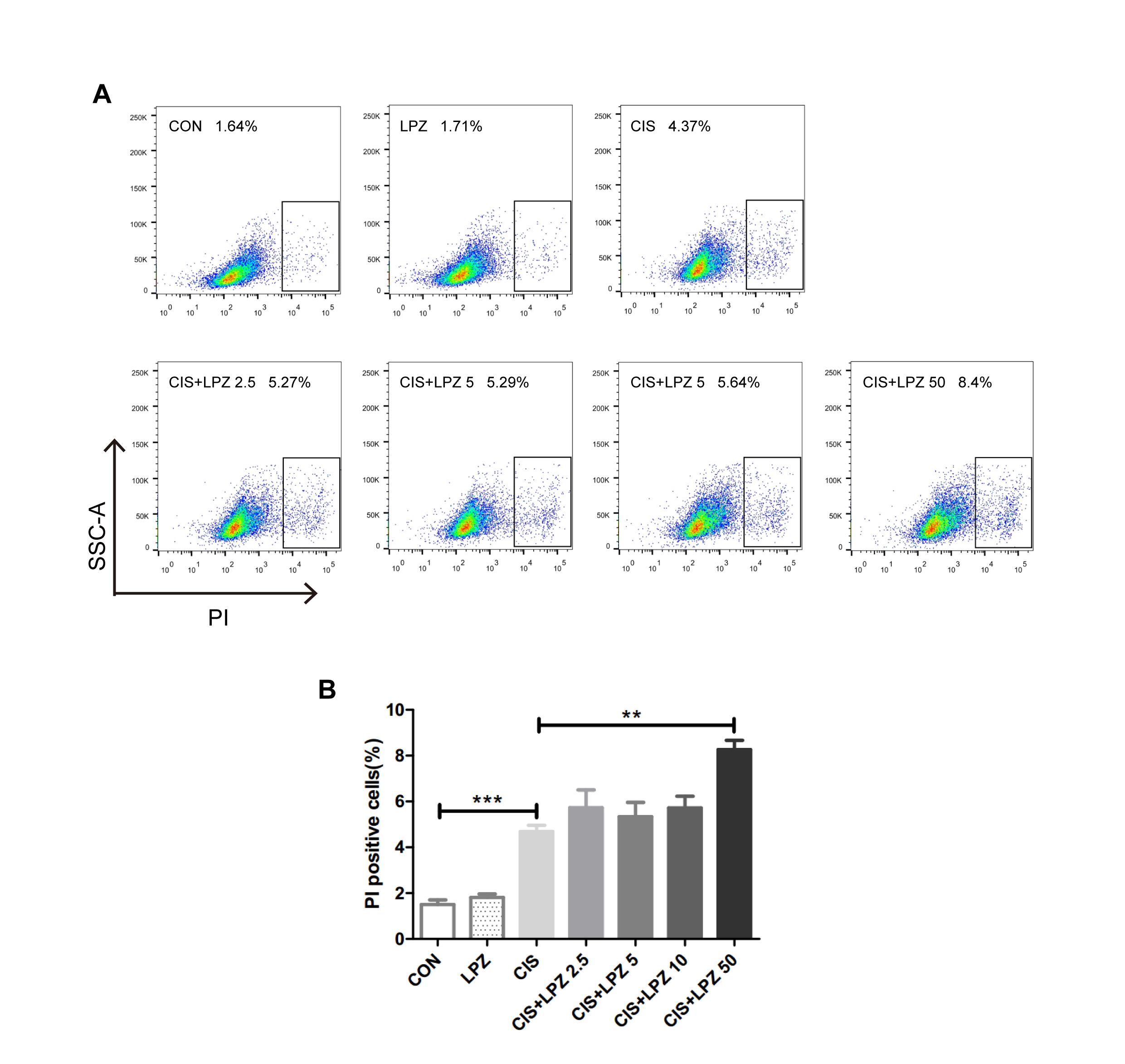

Supplement: Supplementary file 1 — Figure S1 [file JCMM-25-2703-s001.tif]

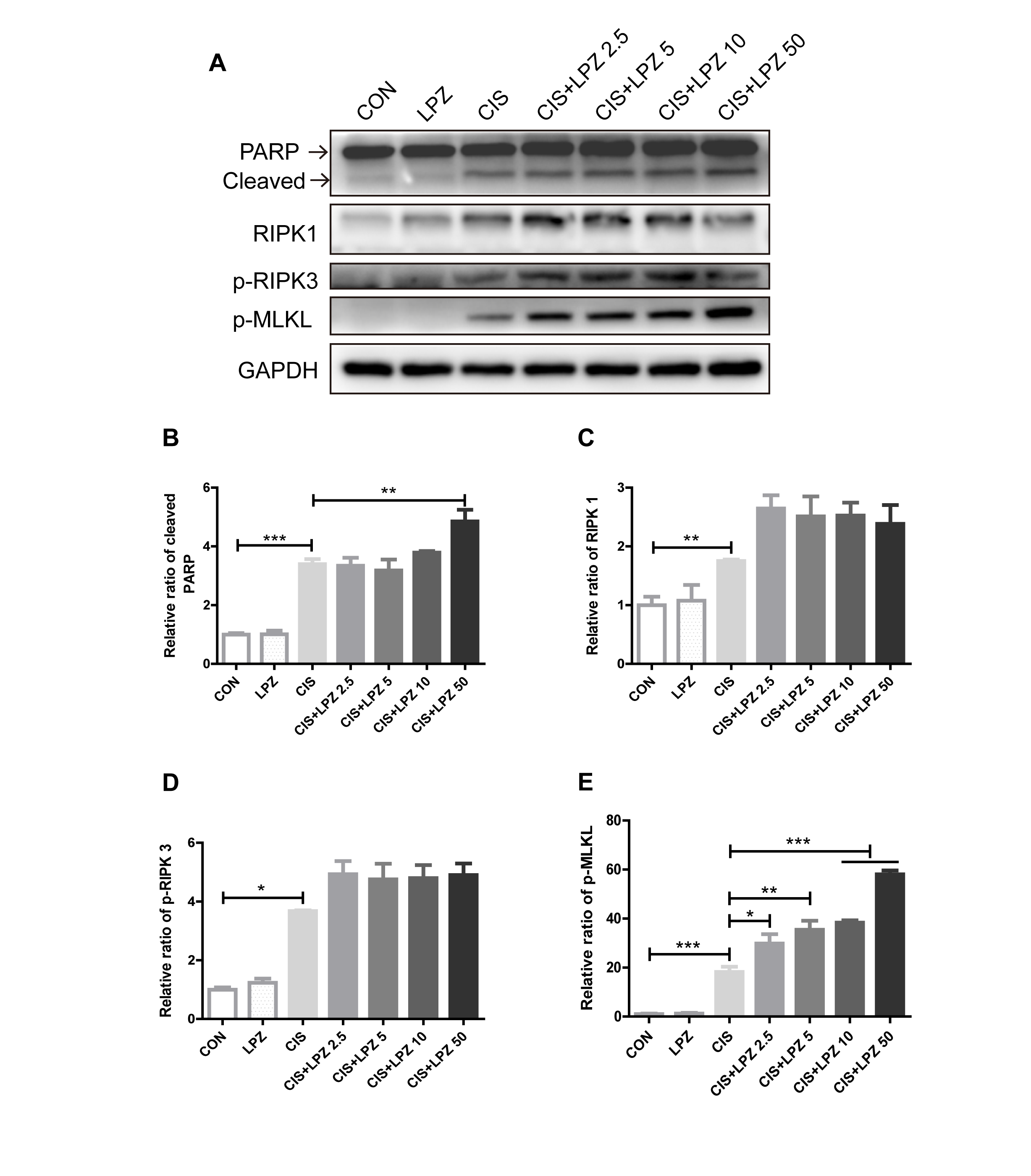

Supplement: Supplementary file 2 — Figure S2 [file JCMM-25-2703-s002.tif]

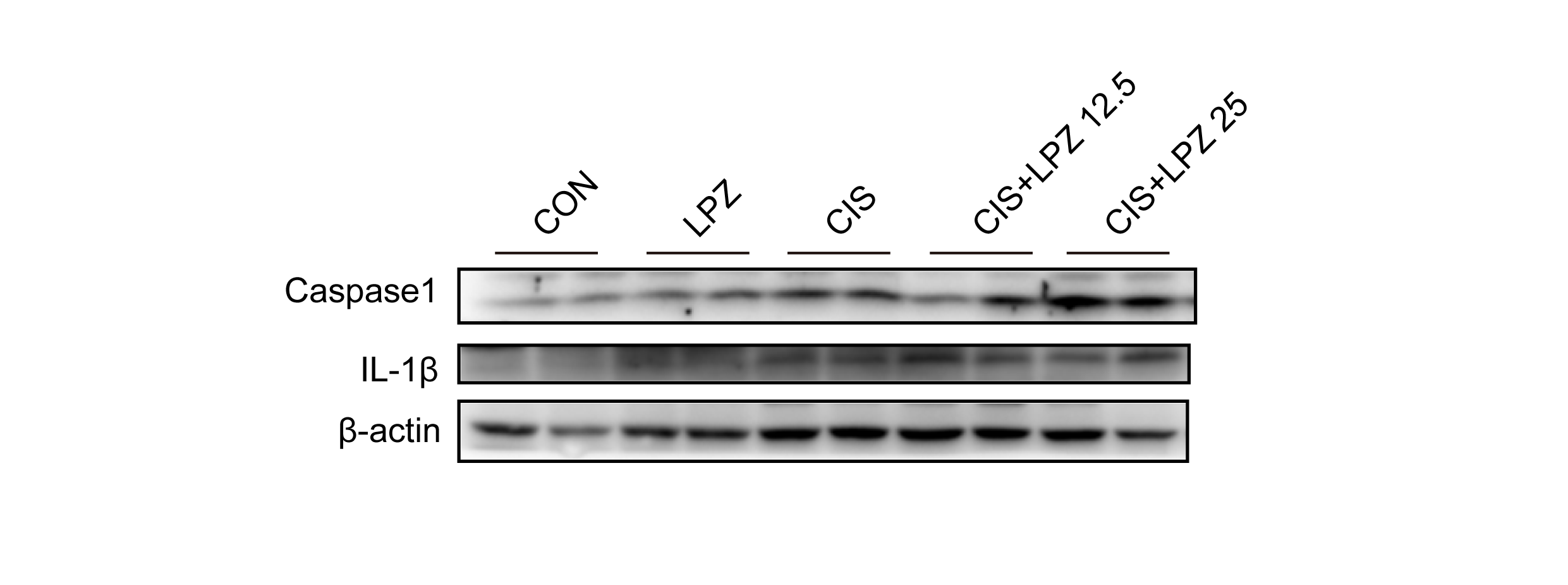

Supplement: Supplementary file 3 — Figure S3 [file JCMM-25-2703-s003.tif]

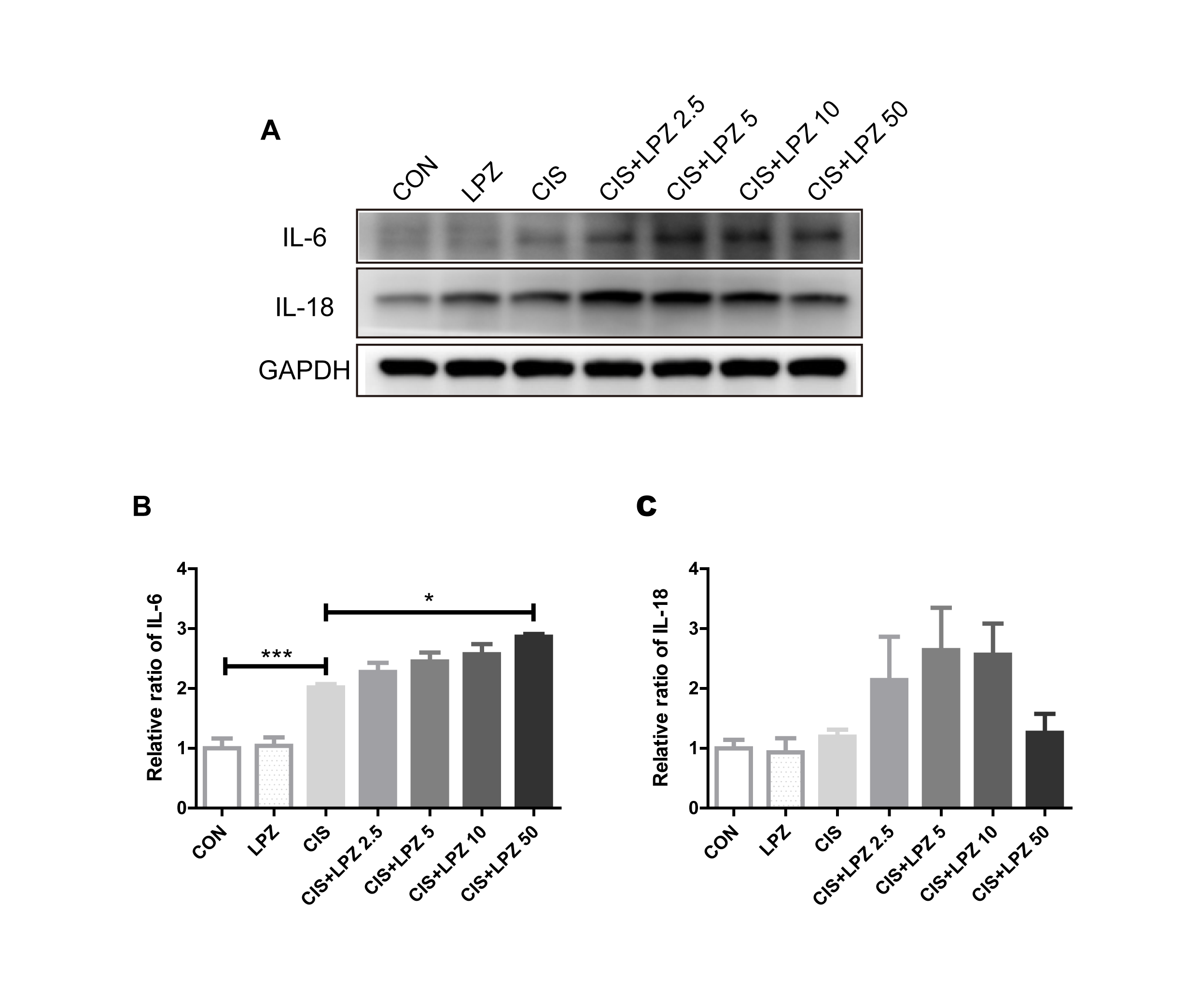

Supplement: Supplementary file 4 — Figure S4 [file JCMM-25-2703-s004.tif]

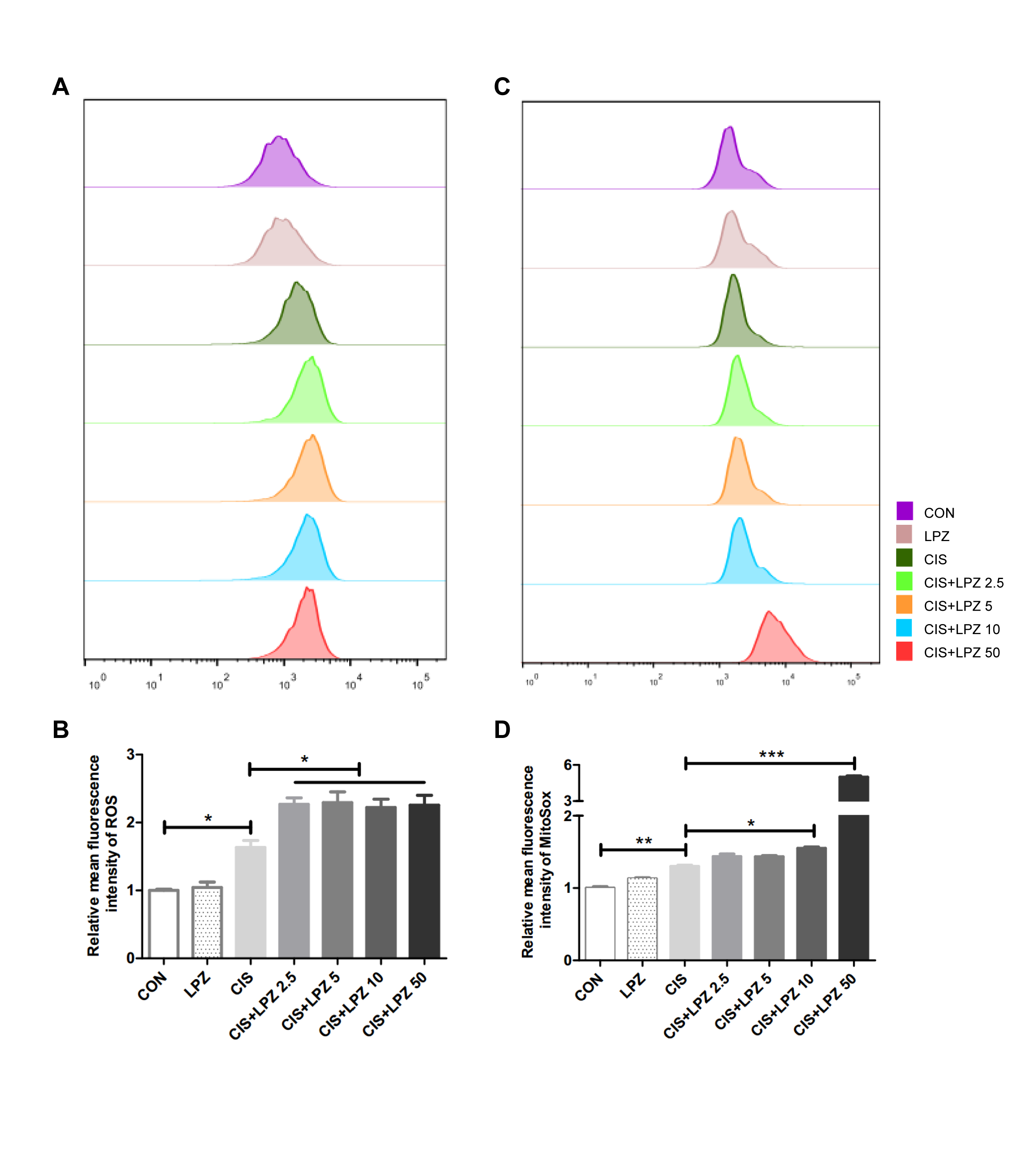

Supplement: Supplementary file 5 — Figure S5 [file JCMM-25-2703-s005.tif]
