# Supplementary material for: Lansoprazole promotes cisplatin‐induced acute kidney injury via enhancing tubular necroptosis
Source: J Cell Mol Med. 2021 Feb 18;25(5):2703–13. doi: 10.1111/jcmm.16302 (PMC7933939; doi:10.1111/jcmm.16302)
Supplement: Supplementary file 6 — Supporting material [file JCMM-25-2703-s006.docx]

**Supplemental Figure Legends:**

Figure S1 Lansoprazole increases HK-2 cell death induced by cisplatin. (A) HK-2 cells were exposed to cisplatin at 80μM with or without different doses of lansoprazole (LPZ) for 24h. After stimulation, HK-2 cells were collected for PI stain. Percentage of PI positive cells was determined by flow cytometry. (B) Quantification of PI positive cells in each group. Results are expressed as mean ± standard error (SE). All data of each group are analyzed using one-way analysis of variance (ANOVA). **, P < 0.01; and ***, P < 0.00, n = 5.

Figure S2 Lansoprazole aggravates HK-2 cell necroptosis induced by cisplatin. (A) Western blot analysis of necroptosis associated proteins, including PARP-1, RIPK1, p-RIPK3, and p-MLKL. GAPDH was used as a loading control. (B-E) Quantification of PARP-1, RIPK1, p-RIPK3, and p-MLKL protein. Results are expressed as mean ± standard error (SE). All data of each group are analyzed using one-way analysis of variance (ANOVA). *, P < 0.05; **, P < 0.01; and ***, P < 0.001, n >=3.

Figure S3 Caspase1 and IL-1β detected by western blot in mice renal tissue.

Figure S4 Lansoprazole exacerbates inflammation induced by cisplatin. (A) Western blot analysis of necroptosis associated proteins, including PARP-1, RIPK1, p-RIPK3, and p-MLKL. GAPDH was used as a loading control. (B-E) Quantification of PARP-1, RIPK1, p-RIPK3, and p-MLKL protein. Results are expressed as mean ± standard error (SE). All data of each group are analyzed using one-way analysis of variance (ANOVA). *, P < 0.05; and ***, P < 0.001, n >=3.

Figure S5 Lansoprazole enhances ROS induced by cisplatin. (A) Overlay of representative flow cytometric histograms of ROS. (B) Quantification of mean fluorescent of ROS. (C) Overlay of representative flow cytometric histograms of MitoSox. (D) Quantification of mean fluorescent of MitoSox. Results are expressed as mean ± standard error (SE). All data of each group are analyzed using one-way analysis of variance (ANOVA). *, P < 0.05; **, P < 0.01; and ***, P < 0.001, n >=3.

**Supplemental materials:**

p-MLKL([ab187091](https://www.abcam.cn/mlkl-phospho-s358-antibody-epr9514-ab187091.html), Abcam, USA), PARP-1 (F2) (sc-8007, Santa Cruz, USA), IL-6 (sc-130326, Santa Cruz, USA), IL-18 ([ab71495](https://www.abcam.cn/mlkl-phospho-s358-antibody-epr9514-ab187091.html), Abcam, USA), Caspase 1 (ab179515, Abcam, USA), IL-1β (AF401, R&D systerm, USA), GAPDH (60004-1-Ig, proteintech, China), MitoSOX Red (M36008, Molecular Probes, USA), CM-H2DCFDA (C6827, Life Technologies, USA), Annexin V, FITC Apoptosis detection Kit (AD10, DOJINDO, Japan).

**Supplemental methods:**

**Cell culture and treatments**

Human proximal tubular HK-2 cells (ATCC) were grown in DMEM (GIBCO, Grand Island, NY) containing 10% FBS under standard conditions. HK-2 cells were exposed to different doses of lansoprazole (L8533, sigma, Germany) at 2.5μM, 5μM, 10μM and 50μM and co-treated with cisplatin at 80μM for 24h. Western blotting was performed to examine the expression of RIPK1, p-MLKL, p-RIPK3, PARP1, IL-6, and IL-18.
